# How Do Electric Fields Coordinate Neuronal Migration and Maturation in the Developing Cortex?

**DOI:** 10.3389/fcell.2020.580657

**Published:** 2020-09-24

**Authors:** Vera P. Medvedeva, Alessandra Pierani

**Affiliations:** ^1^Imagine Institute of Genetic Diseases, Université de Paris, Paris, France; ^2^Institute of Psychiatry and Neuroscience of Paris, INSERM U1266, Université de Paris, Paris, France

**Keywords:** cerebral cortex, development, electric field, neuronal migration, dendritogenesis

## Abstract

During development the vast majority of cells that will later compose the mature cerebral cortex undergo extensive migration to reach their final position. In addition to intrinsically distinct migratory behaviors, cells encounter and respond to vastly different microenvironments. These range from axonal tracts to cell-dense matrices, electrically active regions and extracellular matrix components, which may all change overtime. Furthermore, migrating neurons themselves not only adapt to their microenvironment but also modify the local niche through cell-cell contacts, secreted factors and ions. In the radial dimension, the developing cortex is roughly divided into dense progenitor and cortical plate territories, and a less crowded intermediate zone. The cortical plate is bordered by the subplate and the marginal zone, which are populated by neurons with high electrical activity and characterized by sophisticated neuritic ramifications. Neuronal migration is influenced by these boundaries resulting in dramatic changes in migratory behaviors as well as morphology and electrical activity. Modifications in the levels of any of these parameters can lead to alterations and even arrest of migration. Recent work indicates that morphology and electrical activity of migrating neuron are interconnected and the aim of this review is to explore the extent of this connection. We will discuss on one hand how the response of migrating neurons is altered upon modification of their intrinsic electrical properties and whether, on the other hand, the electrical properties of the cellular environment can modify the morphology and electrical activity of migrating cortical neurons.

## Introduction

Construction of the nervous system is achieved through a complex succession of developmental processes. Among them, two are known to predominantly occur at different developmental stages in the cerebral cortex, cell migration largely embryonically and synaptogenesis postnatally.

Research on chemical cues and adhesion molecules guiding neuronal migration, shaping tissue architecture and synapse formation has shed light on the molecular mechanisms underlying migration as well as morphogenesis of dendrites and spines and have been extensively reviewed (Solecki, 2012; Arai and Pierani, 2014; Barber and Pierani, 2016; Ledda and Paratcha, 2017; Chighizola et al., 2019; Lanoue and Cooper, 2019). Besides molecular mechanisms, electric field (EF) is another factor which has been shown to define the morphology and specification of whole tissues and can in certain cases outplay chemical guidance (McCaig et al., 2009; Levin et al., 2017). The field of cortical development is starting to recognize the importance of ion flow for regulation of early neuronal development: proliferation, migration and differentiation. While nowadays EF is an emerging player in guiding orientation and speed of migrating neurons and in regulating neuronal morphology, it remains to be determined whether and how neuronal migration and morphology establishment are linked.

EFs naturally occur in tissues as a consequence of polarized ion transport inside and outside the cells. Numerous examples of cellular and tissue behavior controlled by bioelectric states are described in amphibians and worms, where altered morphogenesis of whole organs and body parts can occur under ectopic electric stimuli, as well as in mammals during the processes of wound healing, cell proliferation and nerve growth stimulation. Many neural cell types manifest electrotactic behaviors, including neural crest cells and hippocampal neurons (McCaig et al., 2005; Yao et al., 2008; Iwasa et al., 2017). Human neural stem cells migration is also directed by EF while blockade of receptors to classical chemotactic cues does not affect electrotactic responses (Feng et al., 2012, 2017). These results raise the intriguing possibility that cell migration in the developing brain occurs through tissues with steady electrical signals (McCaig et al., 2009; Iwasa et al., 2017) and is guided by them (Li et al., 2014; Feng et al., 2017).

In general, the effects of applied EF on neuronal cells are similar and include changes in length and orientation of cell bodies and leading processes, neurite branching and stimulation of directed migration (Yao and Li, 2016; Bertucci et al., 2019). Indeed, electric stimulation seems to drive all kinds of polarized responses. *In vitro* applications of electric currents to cultured hippocampal cells initiate the cascade of morphological and molecular events. The division cleavage plane turns orthogonally and the mitotic spindle parallel to the EF vector. The Golgi apparatus and centrosome, MAP2^+^ (dendrite-specific) microtubules and eventually the leading process turns to the cathode and cells migrate in a directed fashion with a leading process at the front (Yao et al., 2009). Examples of EF-induced changes specifically in cortical neurons have been reported: cortical axon length and orientation are a subject to specific electric regulation (Tang-Schomer, 2018). Furthermore, electric stimulation of postnatal prefrontal cortical neurons in culture improves dendritic branching and length as well as synaptic protein amounts in both WT and genetically modified conditions (NRG1-knock-out and DISC1-locus impaired mice), associated with psychiatric disorders (Zhang et al., 2017). This provides, on the one hand, a proof for direct electric regulation of cortical dendrito- and synaptogenesis, and on the other hand, an example of electric cue overriding genetic state.

In adult neural tissue, electrical communication is granted through chemical synapses *via* neurotransmitters, which regulate ion flow through ionotropic receptors. Synaptic connections are canonically at the origin of presynaptic Ca^2+^ influx in response to action potential membrane depolarization and post-synaptic in response to neurotransmitter receptors activation. The same machinery is utilized during post-mitotic neuronal migration and maturation: voltage- and neurotransmitter-gated ion channels are capable of regulating the resting membrane potential, which is usually low in immature neurons (Levin et al., 2017). Neurotransmitters are present throughout developing neural tissues, can be released by cells in close vicinity, i.e., neuroblasts or maturing neurons, and can act on migrating neurons in paracrine, non-synaptic, fashion (Spitzer, 2006; Luhmann et al., 2015; Ojeda and Ávila, 2019). Gap junctions, or electric synapses, undoubtedly also play roles during development, especially in electrically active zones, such as the subplate (SP) (Luhmann et al., 2018; Singh et al., 2019).

Here, we aim to analyze EF-guided migration and maturation in the developing cerebral cortex, with a major focus on radially migrating glutamatergic neurons. We use the term EF to designate a sum of electric currents in the tissue and extracellular environment in general as well as electric activity and responses locally, inside the cell.

Dendritogenesis normally occurs after neurons have completed their migration and is, thus, a post-migratory step of neuronal maturation. In the cortical tissue dendritic development is shaped by extrinsic regulation in destined cortical layers (Martineau et al., 2018). Yet, upon electric activity amplification in migrating cortical neurons, precocious and ectopic dendritogenesis is observed (Bando et al., 2016; Hurni et al., 2017). Here we will review the mechanisms mediating the EF-dependent control of neuronal migration and maturation and we will also touch upon how these two processes can be related to synaptic organizing molecules prior to synaptic formation.

## Ca^2+^ Is an Intracellular Proxy of Extrinsic Ef Fluctuations in Developing Neurons

EF-triggered receptors displayed on the cell surface activate a number of signaling pathways, such as ERK, PI3K and small Rho GTPases (Yao and Li, 2016). However, the central regulator of neuronal EF-guided processes, both migration and dendritogenesis, is attributed to downstream intracellular Ca^2+^ concentrations, which convert electrical signaling to physiological responses and are used as a readout of electrical activity (Uhlen et al., 2015; Horigane et al., 2019).

In addition to intracellular Ca^2+^ release in migrating neurons, Ca^2+^ enters from the extracellular environment and is mediated by VGCC type Ca^2+^ channels. These channels are sensitive to membrane depolarization and are typically reactive to synapse-triggered action potentials. In young neurons devoid of synapses, these channels are hypothetically capable of responding to low voltage changes (Horigane et al., 2019). The latter can be produced by neurotransmitter- and voltage-gated ion channels, which are well expressed in the developing cortex and are extensively documented as controlling migration and neuritogenesis in cell-autonomous and non-autonomous ways. Clinical importance of ion channels in early brain development is recognized and indicates their role in transmembrane voltage regulation and/or in migration before stable synapse formation (Smith and Walsh, 2020).

Dendritogenesis in general is very sensitive to extrinsic cues and the molecular mechanisms are well studied and summarized in several excellent reviews (Arikkath, 2012; Valnegri et al., 2015; Ledda and Paratcha, 2017; Lanoue and Cooper, 2019). In cortical neurons, extracellular Ca^2+^ influx is important for dendritic branching, while intracellular Ca^2+^ release affects dendritic branching, axonal growth and density of filopodia (Ramakers et al., 2001). Ca^2+^ events in cortical neurons are localized and may organize dendritic and spine morphology from within: Ca^2+^ waves initiate at dendritic branch points and propagate predominantly at primary apical dendrites. Earlier in development Ca^2+^ events in dendrites are characterized by bigger amplitudes and seem to be dependent mostly by changes of membrane voltage and L type VGCC Ca^2+^ channels (Ross, 2012). Overall, Ca^2+^ signaling in dendritogenesis is well recognized (Konur and Ghosh, 2005).

Intracellular Ca^2+^ fluctuations could thus constitute a convergence point for chemical cues-signaling pathways and EF, summing up in local Ca^2+^ changes that in turn regulate migration, dendritogenesis, and spine formation.

## Cerebral Cortex and Electrically Active Zones

### Direct Studies of EF in the Cortex

In the cerebral cortex, first measurements of tissue endogenous electric current flow were performed in 2013 (Cao et al., 2013) in the walls of the lateral ventricle along the rostral migratory pathway in adult mice. An electric potential gradient measured in the interstitial space along the pathway is of 3–5 mV/mm. It is formed by positively charged ions in the extracellular space, which in turn is supported by currents through cells and tissues. These currents depend on polarized expression of electrogenic pumps (e.g., Na^+^/K^+^ -ATPases), which are effectively suppressed by selective inhibitors with the subsequent reduction of electric currents in the tissue. Neuroblasts migrating along the pathway rise their migration speed or change direction upon application to the brain slice of higher EF or reversal of the field polarity (Cao et al., 2013). Moreover, pharmacological inhibition of EF effectively suppresses migration in 3D cultures of subventricular zone (SVZ) explants, while EF stimulation, in addition to promoting migration, induces expression of adhesive molecules thus increasing cell-cell contacts (Cao et al., 2014).

Evidence for EF-guidance in cortical tissue *in vivo* are coming from the brain injury field. Increased cell proliferation in the SVZ and directed migration toward the source of the current is observed during motor cortex electrical stimulation (Jahanshahi et al., 2013). Human neural stem cells transplants into the rat rostral migratory stream are efficiently guided by endogenous EF, while applied electric stimulation redirects migration of subpopulation of cells regardless of endogenous tissue cues (Feng et al., 2017).

### Electrical Activity During Cortical Development

There are no studies on applied or otherwise manipulated direct EF specifically for the developing cortex and migrating neurons in early stages. Nevertheless, electrical activity of migrating cells in the embryonic cortex is well documented. Individual neurons display spontaneous Ca^2+^ activity (Corlew et al., 2004; Egorov and Draguhn, 2013; Bando et al., 2016; Yuryev et al., 2018; Mayer et al., 2019) and inward Na^+^/outward K^+^ currents (Picken Bahrey and Moody, 2003). Ca^2+^ waves through the mouse cortical plate are recorded *in utero* (Yuryev et al., 2016). Recordings in the human developing subplate show spontaneous oscillatory activity of GABAergic origin, but almost no synaptic connections (Moore et al., 2011).

**FIGURE 1:**
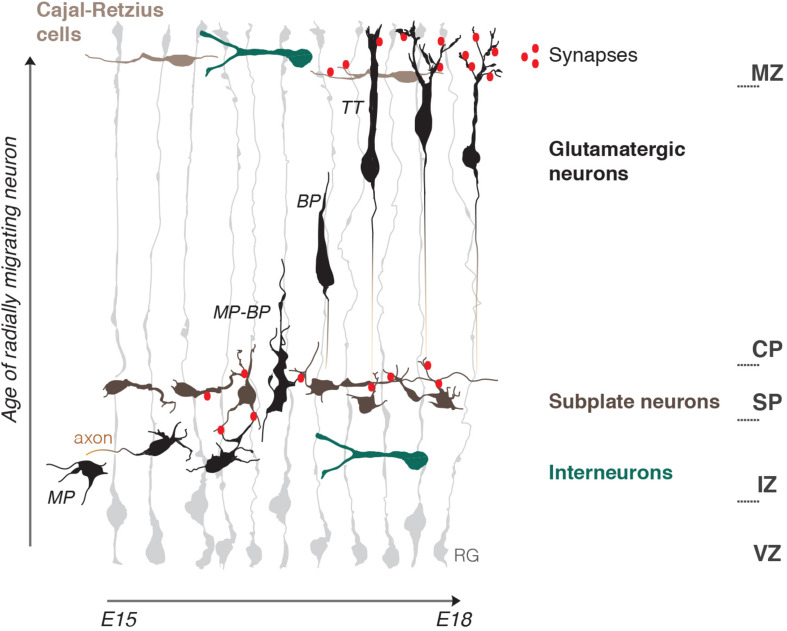
Schematic representation of the embryonic mouse cortex and its electrical zones. Two electrically active borders, the subplate (SP) and the marginal zone (MZ), organize neuronal migration in the developing cerebral cortex. The MZ and SP coincide with morphology and migration mode transformations of radially migrating neurons and represent pathways for tangentially migrating neuronal populations: Cajal-Retzius cells and Interneurons. Note the presence of early functional synaptic contacts in the SP and MZ. Axonogenesis and polarization of migrating neurons occur under the SP and dendritogenesis in the MZ. Neuritogenesis is thus enhanced within the two zones. Radial migration is depicted starting from bipolar neurons (BP) in the intermediate zone (IZ) onwards that represent steps mostly studied in terms of electrical activity. RG, radial glia; MP, multipolar neuron; BP, bipolar neuron; MP-BP, transitional morphology of the neuron going through the SP; TT, neuron undergoing terminal translocation; VZ, ventricular progenitors zone; IZ, intermediate zone; CP, cortical plate.

The developing cerebral cortex is a rapidly expanding layered structure that mostly relies on highly organized radial migration of newly born glutamatergic neurons. During radial migration neurons undergo morphological changes which are accompanied by the change of migration mode and are influenced by tissue environments (Ohtaka-Maruyama and Okado, 2015; Figure 1).

Just born multipolar neurons slowly migrate toward the pial surface through the intermediate zone (IZ) by a multipolar migration mode, with long pauses and frequently changing directionality (Tabata and Nakajima, 2003). This continues until they reach the first structural «border», the SP. The SP is a layer of transient electrophysiologically active and morphologically mature neurons which manifest a high electrical activity as they establish afferent and efferent synaptic connections within the developing cortex (Luhmann et al., 2018). Here multipolar neurons start their polarization process by the formation of tangentially oriented axonal outgrowth (Hatanaka and Yamauchi, 2013). This is well-studied from a biochemical point of view. Reelin, a powerful chemical regulator of radial migration, is present in the IZ and, by initiation of a RAP1-dependent N-cadherin cell surface rise, allows multipolar neurons to sense microenvironmental cues, which in turn can induce polarization (Hansen et al., 2017). Axonal induction is also promoted by GABA_B_ receptor, and GABA is believed to be present in the IZ due to tangentially migrating interneurons (Bony et al., 2013) (also see *GABA* chapter). Moreover, SP neurons make glutamatergic synaptic contacts with multipolar migrating neurons. This induces NMDAR-dependent Ca^2+^ entry into the migrating neuron and, as a result, facilitates the morphology and migration mode switch to bipolar neurons (Ohtaka-Maruyama et al., 2018; Ohtaka-Maruyama, 2020). Bipolar neurons then migrate by locomotion along radial glia fibers. It is possible that electric stimulation from radial glia is adding to the multipolar-bipolar transition, as Ca^2+^ bursts in both cell types are synchronized during this process (Rash et al., 2016).

Bipolar locomotion is a fast mode of directed migration in which speed and pausing time is managed by the strength of intracellular Ca^2+^ transients (Hurni et al., 2017). The final stage of migration is characterized by a change to terminal translocation, rise of intrinsic frequencies of Ca^2+^ transients and appearence of dendrites, a signature of mature post-migratory neurons (Bando et al., 2016; Hurni et al., 2017). Neuronal dendrites spread out to the marginal zone (MZ), the upper limit «border» for radial migration. The MZ is a low cell density layer at birth, and, as the SP, develops rather early synaptic connections due to a local population of more mature Cajal-Retzius neurons (CRs) (Janušonis et al., 2004) and young post-migratory neurons (Bouwman et al., 2004).

CRs are early born tangentially migrating neurons which are best known for Reelin expression and its role as a regulator of terminal translocation and dendritogenesis (O’Dell et al., 2015; Hirota and Nakajima, 2017). CRs precise number and distribution have refined functions in cortical circuits organization. Thickness of apical dendritic tufts and of the MZ depend on CR density, as well as the excitation/inhibition ratio of post-migratory neurons. CRs migration is dependent on NMDARs stimulation and therefore is also activity-dependent (de Frutos et al., 2016; Riva et al., 2019). Two key chemical regulators of multipolar-bipolar and terminal translocation steps, Reelin and Dab1 (Hirota and Nakajima, 2017; Zhang et al., 2018) are surprisingly upregulated by electromagnetic field exposure (Hemmati et al., 2014). Altogether these data suggest that electric currents may be well upstream of chemical regulation of radial neuronal migration.

### Is There EF in the Developing Cortex?

The presence of an electrically active boundary during cortical development, which organizes neuronal migration was described by Ohtaka-Maruyama (2020). It is possible to further imagine the developing cortex as a stratified structure of variable electric strength. For instance, the SP and the MZ are possibly highly charged compared to the relatively low EF in the IZ and the cortical plate (CP). They, thus, both could serve as electrical guide borders which, together with chemical cues, help attracting migrating glutamatergic neurons in the direction of the pia, orient their polarization and eventually drive corresponding morphological changes: axon initiation under the SP and dendritogenesis in the MZ.

How could this be exerted mechanistically on the migrating cell? As explained by Yao and Li (2016), when a cell is submitted to EF, due to a large membrane resistance, ionic flow is forced mainly around the cell. This creates extracellular current along the cell sides and lateral voltage gradient along the upper and lower membrane surfaces. Charged lipids and proteins, including conductance channels, are redistributed by the electrophoretic force, and form clusters. Voltage-gated ion channels could respond directly, creating local differences in resting membrane potentials and subsequent stimulation of Ca^2+^ influx and signaling activation. For example, increase of the Ca^2+^ influx on one side may signal the cell to form localized lamellipodia (Yao and Li, 2016). The same principle would apply to neurotransmitter-gated ion channels in the presence of neurotransmitter gradients, as discussed in the chapters below.

Some data from related models could support this view. Cao et al. (2014), have shown that EF gradients are present in cultures of SVZ explants from postnatal mice. In their study neuroblasts migration without growth factors is random and cells have multipolar morphologies, but when EF of physiological strength is applied, cells acquire a bipolar morphology and the directionality of migration significantly increases (Cao et al., 2014) – a situation remarkably similar to the multipolar-bipolar switch in the SP. A wealth of studies on the behavior of neural stem cells upon electric stimulation has established an *in vitro* model that remarkably reminds of the developing cortex organization in the radial axis: in the absence of electric stimulation stem cells self-renew or differentiate into neurons or astrocytes and oligodendrocytes. After EF application they proliferate more efficiently with a shift toward neurons, cells become polarized, migrate toward the cathode and show an increase in intracellular Ca^2+^ (Bertucci et al., 2019). These observations, collected upon direct electric stimulation on immature neurons, in the absence of chemical stimuli, accurately reproduce complex behaviors of intact young neurons in the developing cortex tissue, and therefore suggest that EF variations are capable of inducing a whole panel of elaborated migration and maturation behaviors of glutamatergic cortical neurons.

## Neurotransmitters, Their Receptors and Ion Channels

Migration and dendritogenesis are processes separated both spatially and temporally, suggesting specific mechanisms of regulation. This is true for both tangentially and radially migrating populations. There is a plethora of data on electric modification of cortical neurons leading to migration delays with or without further defects in dendritogenesis. In the following sections, we attempt to dissect possible mechanisms underlying the electric control of migration and dendritogenesis by correlating ion channels distribution and their known effects on these two processes.

### Glutamate and Its Receptors

Ambient glutamate concentration in the neonatal cortex is high compared to later postnatal stages, when it is likely uptaken by astrocytes (Hanson et al., 2019). Since neuronal migration and differentiation occurs prior to astrocytic differentiation, which starts at end of the embryonic period, extracellular glutamate concentrations may be even higher at prenatal stages. The source of extracellular glutamate is not exactly known. It can be released when vesicular neurotransmission is blocked and it has been suggested that it acts in a paracrine manner and may be sequestered around migrating neurons (Luhmann et al., 2015). Glutamate has been shown to act as a chemoattractant of cortical neurons *in vitro* (Behar et al., 1999).

**FIGURE 2:**
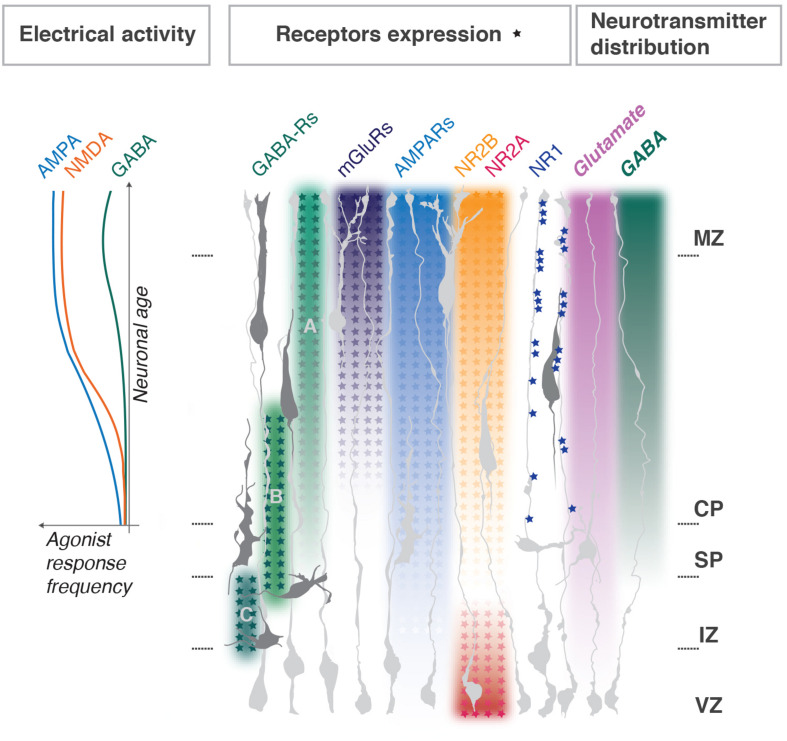
Distribution of neurotransmitters and their receptors during cortical development. Enrichment gradients of ion channels and their specific subunits as neurons develop correlate with Ca^2+^ responses to corresponding agonists (graph to the left, adapted from Mayer et al., 2019) and dendritogenesis in the post-migratory neurons. NMDA NR1 subunits clustering in radial glia fibers regulate neuronal layer positioning; NMDA NR2A and B expression switches as progenitors differentiate into neurons. Metabotropic glutamate receptors gradients are representative for mGluRs1, 3, 4, 5, and 8; for AMPARs – GluR1, 2, 3, 4. GABA_A_, GABA_B_, and GABA_C_ receptors functional switches in radially migrating neurons are shown. MZ, marginal zone; CP, cortical plate; SP, subplate; IZ, intermediate zone; VZ, ventricular progenitors zone. Major references Hurni et al. (2017), Mayer et al. (2019), and Pasquet et al. (2019).

Glutamate receptors, namely NMDARs, AMPARs and mGluRs, are expressed in the developing cortex. However, their subunit distribution throughout the developing cortex is uneven and in some cases, they display a clear developmental switch (Luhmann et al., 2015; Mayer et al., 2019; Figure 2).

Many studies on the role of NMDARs and AMPARs in migration are summarized in excellent reviews (Rakic and Komuro, 1995; Luhmann et al., 2015; Horigane et al., 2019; Ojeda and Ávila, 2019). In general, NMDAR and AMPAR blockade attenuates the migration of different types of immature neurons and NMDA physiological activation accelerates the movement. Selective manipulations of both NMDARs or AMPARs subunits by genetic and acute invalidation, however, do not exactly reproduce these effects in radially migrating glutamatergic neurons of the developing cortex, leaving the interpretation still unresolved.

#### NMDARs Subunits

NR1 (a.k.a. Grin1 or GluN1) is absent from the progenitor zone and present in the CP. It is an essential subunit for NMDAR function, but surprisingly its manipulation does not always lead to dramatic alterations in the developing cortex. Luhmann et al. (2015) review a series of studies on cell-type-specific NR1 knockouts (one good example is Iwasato et al., 2000), which indicate either the existence of compensatory mechanisms or extrinsic regulation of migration by non-neuronal target structures, like glial cells. One recent study supports the latter (Pasquet et al., 2019). Here, the authors demonstrate that proper layering of radially migrating neurons relies on NR1 clustering in radial glia fibers at contact sites with the soma and leading process of bipolar neurons (Figure 2). On the other hand, acute NR1 downregulation (KD) by *in utero* electroporation (IUE) into neuronal progenitors severely delay the migration of electroporated cells, without changing neuronal fate determination (Jiang et al., 2015). Measurements of dendritic structure of NR1 KD neurons located ectopically in lower cortical layers showed a simplistic morphology, as did controls (siRNA for a motility agent). However, KD neurons which reached the proper layer still showed a simplistic morphology (Jiang et al., 2015). Therefore, NR1 correct expression mediates proper dendritogenesis of post-migratory cortical neurons. Overall it is difficult to uncouple the role for NR1 in both processes. Furthermore, its cell-autonomous role in migrating neurons remains debatable.

NR2A and B (a.k.a. Grin2A,B or GluN2A,B) are modulatory subunits and often undergo a developmental switch in various neural tissues, including the cerebral cortex (Figure 2). The switch sharpens the response to glutamate as it yields channels with faster kinetics which are important for regulation of maturation of neuronal circuits (Mayer et al., 2019). In agreement with the expression pattern, NR2B, but not NR2A, cell-autonomous downregulation impedes radial migration, without changes of neuronal identity. Neurons aberrantly located in lower cortical layers develop dendritic trees of abnormally high complexity, and those neurons which reach the targeted position are comparable to controls (Jiang et al., 2015). Therefore, it is possible that NR2B function in physiological dendritogenesis is related to inhibition of the process. Based on these studies it is hard to uncouple its specific roles in dendritogenesis *vs.* migration.

Recent reports further highlight a substantial role for NR2B in neuronal maturation, dendritogenesis and non-synaptic NMDAR function. Human neural progenitors carrying autism spectrum disorder (ASD)-associated NR2B variants show impaired Ca^2+^ influx, membrane depolarization and differentiation failure (Bell et al., 2018). Most of NR2B-containing receptors are found within dendrites and the cortical neurons carrying ASD-associated variants manifest less dendrites, shorter total length and overall dysmorphia, while spine density or morphology is not altered. Mechanistically these mutations abolish channel activity and show no surface expression and reduced delivery to neurites (Sceniak et al., 2019). A more refined mechanism is proposed by a study on hippocampal neurons and cortical spiny stellate cells where dendritic length regulation and branching are uncoupled, with only the latter relying on NR2B (Espinosa et al., 2009).

It is interesting to note that among the known developmental NMDAR-dependent channelopathies it is exactly NR1 and NR2B gain of function variants which cause early developmental migration phenotypes, such as polymicrogyria (Smith and Walsh, 2020). This suggests that gain of function mutations might primarily affect migration in human developmental pathologies.

#### AMPARs and Metabotropic Glutamate Receptors

AMPA receptors expression increases in cortical neurons throughout development (Hurni et al., 2017; Mayer et al., 2019; Figure 2) and, similarly, to NMDARs, have been involved in both migration and dendritogenesis. Pharmacological studies with the AMPA/kainate receptor antagonist CNQX reveal the role in motility dynamics of migrating neurons; enhanced stalling and directionality changes are explained by lack of coordination between soma and leading process extension, possibly due to problems with growth cone dynamics (Jansson et al., 2013). It is important to mention, however, that CNQX is not exclusively selective for AMPARs but acts as well as NMDARs antagonist at glycine site (Lester et al., 1989), so the described effects are hard to dissociate between the two receptors. There is, however, an important argument against a role for AMPARs in radial migration: out of all glutamate receptor ligands only NMDA and L-glutamate (and not AMPA, D-glutamate, kainate or quisqualate) induce chemotactic motility responses in mouse cortical neurons (Behar et al., 1999).

Dendritic arbor development of glutamatergic neurons and interneurons is mediated by distinct AMPA subunits. GluR1, 2 and 3 are involved in dendritogenesis of glutamatergic cortical neurons and their action is associated with spontaneous increase of Ca^2+^ transient amplitudes, but not frequency. Few studies report specifically an increase of dendritic arbor complexity at the third level branches and higher (Chen et al., 2009; Hamad et al., 2014). Others detect strong upregulation of dendritic length by subunits “flip” isoforms particularly enriched in development (Hamad et al., 2011). GluR1 (and not −2 and −3) is more specific for interneuron dendritogenesis (Hamad et al., 2011). Indeed, interneurons, migrating in the IZ, become more rounded after AMPA exposure and this is mediated by paracrine AMPA receptor activation (Poluch et al., 2001). Therefore, GluRs might participate in guiding the migratory stream, or provide stop signals for migrating interneurons and initiate their maturation.

One hypothesis for AMPA-regulated Ca^2+^ dynamics in immature neurons rests on their deficiency in subunits [e.g., GluR2 (GluA2)] which causes cortical neurons to be permeable to Ca^2+^, while their gradual enrichment toward birth strengthens Ca^2+^ influx control (Kumar et al., 2002). This is supported by a study in which activation of Ca^2+^-permeable AMPA receptors induced neural progenitor cells (NPCs) to differentiate to the neuronal lineage and increased their dendritic arbor formation (Whitney et al., 2008). Overall, AMPARs may be a good candidate for preferential regulation of dendritogenesis over the migratory effects on cortical maturing neurons.

AMPARs actions on dendritogenesis interplay with those of metabotropic glutamate receptors, mGluRs (Grms). One example is the disruption of dendritogenesis in mGluR5 knockout cortical neurons associated with an increase of Ca^2+^-permeable AMPA receptors (Huang and Lu, 2018). However, mGluRs also have a role in stalling during neuronal migration, which is believed to be due to highly localized Ca^2+^ changes and is an important part of migration as it may be rising sensitivity to chemical cues, helping direction searching (Jansson et al., 2013). Although not ionotropic, mGluRs activation seems to be linked to triggering Ca^2+^ high-amplitude waves propagation (typical for developing tissues) only in subregions of the dendrites (Ross, 2012), and therefore in interaction with AMPARs they could contribute to fine dendritic organization.

### GABA and Its Receptors

GABA is one of the earliest neurotransmitters expressed in the nervous system and is enriched during early corticogenesis (Lauder et al., 1986; Behar et al., 1996; Bony et al., 2013). GABA influences radial and tangential migration through its various receptors depending on the migration step and exerts some of these actions through Ca^2+^ influx signaling, due to the fact that that it functions as an excitatory neurotransmitter in early development and depolarizes immature neurons. GABA paracrine and chemoattractive actions have been documented by several authors (Horigane et al., 2019; Ojeda and Ávila, 2019).

Glutamatergic neurons migration can be modulated by GABA in a concentration-dependent manner and relies on pharmacologically distinct classes of GABA receptors. VZ/IZ populations, show directed migration in response to femtomolar GABA concentrations. Cells which exit from the proliferative zone to the IZ are blocked by GABA_C_ ionotropic channels antagonist, and GABA_C_-R, which has a high affinity to GABA, delivers a signal that maintains migration throughout the IZ (Behar et al., 1998, 2000; Denter et al., 2010). IZ-CP entry is sensitive to G-protein inhibitors, indicating a role for metabotrophic GABA_B_ G-protein coupled receptors. Neurons migrating in the CP respond to micromolar amounts of GABA with increased cell motility and this response partially relies on G-protein. This is also sensitive to depolarizing agents: glutamate, potassium and GABA_A_ ionotropic channels activation (Behar et al., 1998, 2000). GABA_A_-R regulates migration speed in the upper CP and most of all is important for migration termination before the MZ with GABA tonically reducing the speed of cell migration in the upper cortex *via* GABA_A_-R activation by interfering with Ca^2+^ oscillations (Heck et al., 2007). GABA_A_-R-dependent regulation of migration may also be mediated by taurine, a compound abundantly present in developing tissues (Furukawa et al., 2014). Therefore, there is an elegant model on the regulation of radial migration by GABA concentration gradients through receptor expression switches depending on the physiologic state of the migrating neuron, crossing each developmental zone (Figure 2).

After birth young neurons switch response to GABA from excitatory to canonical inhibitory (Ben-Ari et al., 2012). GABA-induced excitability decreases due to lowering of intracellular Cl^–^ concentrations *via* developmental upregulation of KCC2, a K^+^/Cl^–^ cotransporter extruder, and downregulation of NKCC1, the chloride-inward Na^+^-K^+^-Cl^–^ cotransporter. The GABA_A_-R-dependent Cl^–^ flux reverts and becomes hyperpolarizing (Bortone and Polleux, 2009; Horigane et al., 2019). This mechanism is at the basis of the termination of interneurons migration. Before the switch, ambient GABA and Glutamate signals are motogenic, but once interneurons are in the cortex the decrease of Ca^2+^ transients upon GABA_A_-R activation induces them to stop (Bortone and Polleux, 2009).

For radially migrating neurons excitatory GABA actions exerted through GABA_A_-R are indispensable for morphological maturation. Premature overexpression of KCC2 as well as downregulation of NKCC1 do not perturb migration (Cancedda et al., 2007; Jiang et al., 2015), but have a dramatic effect on dendritic morphology (Ge et al., 2006). KCC2 overexpression prevents GABA-induced Ca^2+^ elevation and the morphological impairment of properly positioned upper layer neurons comprises pronounced reduction of total dendrite length and branch number, with very few dendritic processes projecting to layer 1 (MZ). The effect worsens with time. Experiment with overexpression of the inward-rectifier K^+^ channel Kir2.1 produced similar results, further indicating that reducing membrane depolarization is sufficient to impair dendritogenesis in cortical neurons (Cancedda et al., 2007; Sernagor et al., 2010). However, also direct disruption of GABA_A_ receptor activity without perturbations of cell polarization produces similar effects. DISC1-KD in young cortical pyramidal neurons leads to perturbations of surface expression of the GABA_A_-R subunit, while Cl^–^ cotransporters are unaffected (Saito et al., 2016). Nonetheless, GABA-mediated Ca^2+^ influx is diminished, as demonstrated by GABA_A_-R antagonist treatment. Acute DISC1-KD in postnatal cortical neurons prevents complex dendritic arborization development and this is accompanied by GABA-mediated post-synaptic currents impairments (Saito et al., 2016). Thus, dendritogenesis is mediated by direct GABA_A_ receptor activity as well as by the hyperpolarized state of the neuron.

GABA_B_ metabotropic receptor has been found to be crucial for axon-dendrite polarization and growth. GABA_B_ downregulation in a dose-dependent manner leads to migration delays and ectopic accumulation of cells with long and thin processes, as well as reduced development of dendrites and pronounced axonogenesis in cells reaching the upper CP. *In vitro* GABA_B_-R signaling specifically affected axonal initiation. The mechanisms rely on cAMP-dependent phosphorylation of LKB1, a kinase involved in neuronal polarization (Bony et al., 2013). This goes in concordance with the specific receptor role in the IZ-CP transition, which thus may rely on the multipolar-bipolar transition regulation. In tangential migration GABA_B_ has a pronounced role in a concentration-dependent motility regulation, through regulation of the length of the leading process, and those effects are not accompanied by membrane potential changes, highlighting that mechanism does not rely on electric activity modulation (López-Bendito et al., 2003). Therefore, the primary role of GABA_B_-R is related to neuronal polarization and subsequent directed migration rather than to dendritogenesis.

### Other Ion Channels: Are Migration and Dendritogenesis Uncoupled?

Most studies on neurotransmitter channels do not clearly discriminate whether the effects on dendritogenesis are a direct consequence of those on termination of migration. Few interesting examples below illustrate how ion channels, which modulate electrical activity, can regulate migration and dendritogenesis and possibly help the distinction.

Prokaryotic voltage-gated sodium channel, NaChBac, when overexpressed in cortical radially migrating neurons, dramatically raises excitability and the frequency of spontaneous Ca^2+^ transients. This causes premature migration arrest, but also induce dendritogenesis in ectopic cells. Moreover, the ectopic cells appear to prematurely complete their migration since they loose contact with radial glia fibers. The authors show that migrating neurons have lower Ca^2+^ oscillations parameters than the post-migratory and thus they hypothesize that the difference between neuronal migration and maturation relies on the intensity of spontaneous Ca^2+^ transients (Bando et al., 2016). Another study aiming to stimulate cell-intrinsic activity used an artificial receptor-ligand system (DREADD). The results obtained here were overall similar to those of NAChBAc: cells with raised Ca^2+^ transients frequencies, and not durations, were massively delayed in the IZ-SVZ and lower CP, without changing their birthdate-dependent identity. Moreover, ectopic neurons developed neuritic branching reminiscent of dendrites. The same study, similarly, observed raised Ca^2+^ transients in neurons undergoing migration termination. Moreover, the authors were able to show that DREADD-induced cell migration delays were associated with an increase in pausing time, and not instant migratory speed (Hurni et al., 2017). These studies reinforce the idea that a developmental increase in Ca^2+^ events intensity plays a role in migration arrest and, eventually, maturation.

While the above studies are very illustrative, they depend on artificial expression of non-endogenous channels, which probably overstimulate electrical activity to abnormally high levels and thus cannot exactly reflect the physiological situation in the mammalian cortex. A recent study by Smith et al. (2018) addresses the role of SCN3A, a subunit of voltage-gated sodium channel NaV1.3, which is naturally enriched in migrating neurons of the developing human cortex and is downregulated upon cortical maturation. Few point variants in this gene are associated with rare cases of developmental channelopathies which range from polymicrogyria and intellectual disability to microcephaly and severe seizures. Acute overexpression of the gene and its mutant forms in the ferret cortex highlighted the role in migration and gyrification (additional sulci and gyri) with heterotopic formations exclusively registered in the case of mutants. Overexpression of SCN3A in human cortical neurons promoted dendritic branching and this effect was attenuated in mutant forms. Patch clamping human fetal cortical neurons demonstrated the absence of action potentials, therefore SCN3A likely contribute to Na^+^ conductance that modulate other voltage-dependent processes like Ca^2+^ signaling. Altogether, these data suggest, once again, that dendritic branching and migration effects are closely interconnected. However, it is important to point out that the same variant (F1759Y) which aggravates migration phenotypes (more severe gyrification and heterotopia) also attenuates dendritogenesis which goes in the opposite direction to the postulate that simple neuronal activity overstimulation is ultimately responsible for both processes.

Notably, migration arrest due to enhanced neuronal activation is not always accompanied by dendritogenesis. The KCNK family of leak potassium channels conducts potassium currents at resting membrane potential, with little voltage dependence, and is one of the major determinants of neuronal excitability in the cortex. Family members are expressed throughout the cortex and have a role in radial migration with the most prominent phenotypes observed for KCNK9. KCNK9 downregulation as well as overexpression of mutant forms impair radial migration by increasing the frequency of spontaneous Ca^2+^ transients, possibly by controlling resting membrane potassium permeability. The ectopic delayed cells do not die neither they change their identity, but persist in deeper cortical layers showing undeveloped morphology for a prolonged period of time (Bando et al., 2014). Since the correct positioning of neurons is crucial for proper dendritogenesis (Martineau et al., 2018), these results imply that KCNK9-induced Ca^2+^ transients increase is not sufficient to promote ectopic dendritic morphology development, while it is sufficient for the migration arrest.

These few studies demonstrate that although migration, its final termination and dendritogenesis are intimately connected and likely rely on similar mechanisms of electrical activity rise at the maturation stage, the mode of this electrical activity regulation by ion transport type and intensity may vary: from very intense which causes ultimately cell migration arrest and strong ectopic dendritogenesis (like stimulation with DREADD) to milder which only affects migration (like KD of KCNK9). Moreover, it is possible that channel conformation changes due to mutations contribute to its activity and somehow regulates migration and dendritogenesis in opposing manner, as it seems to happen with SCN3A pathological variants.

## Synaptogenesis and “Synaptic” Proteins

It is generally believed that functional chemical synapses massively appear within the first 2 weeks after birth. However, the exact time of establishment of fully functional synaptic structures is somehow vague. Synapses are complex and comprise many molecules which are supposed to be specific, but have a remarkably variable expression span and, often, functionality (Farhy-Tselnicker and Allen, 2018; Südhof, 2018).

The developing mouse cortex gradually shapes morphologically recognizable synapses. First clearly immature synapses (with pleiomorphic vesicles associating around newly formed terminals, with yet thin pre- and post-synaptic plasmalemmas and narrow gap in between) are identifiable as early as E15 (Li et al., 2010). Functional synapses likely appear in electrically active borders such as the SP and MZ (Figure 1). SP neurons forming full synaptic connections with multipolar migrating neurons were observed at E16 in mice (Ohtaka-Maruyama et al., 2018). These connections are defined (i) morphologically by VGLUT2 staining, and the presence of vesicles and electron-dense structures reminiscent of active zones and post-synaptic densities, and (ii) functionally by the presence of Ca^2+^ transients and exocytosis of presynaptic vesicles at the upper IZ. However, data suggest that these functional synapses may be present earlier (Ohtaka-Maruyama et al., 2018). SP neurons may also send electrophysiologically active GABAergic projections to CR cells in the MZ as seen soon after birth (Myakhar et al., 2011), but synaptogenesis on CRs is described already at E17 (Janušonis et al., 2004).

Morphological synapses in the MZ, which are largely formed by two classes of neurons, are registered as early as E16 in mice. Their ultrastructure satisfies the parameters of morphologically well-formed mature synapses: presynaptic vesicles conglomeration, presence of docked vesicles and active zone. By E18, MZ abundantly expresses a list of structural synaptic proteins, including post-synaptic density marker PSD95, synaptic vesicle associated VAMP2, and AMPA subunits 2 and 3. However, the functionality of these synapses may not be fully established yet: in the absence of neurotransmitter release and, thus, synaptic electric activity (Munc18-1 null mice) these synapses are largely preserved (Verhage et al., 2000; Bouwman et al., 2004). A structurally complete synapse, therefore, does not necessarily mean it is electrophysiologically functional and, in fact, may not require electrical activity to be formed.

Many synaptic molecules are expressed in the developing cerebral cortex prior to full synaptic formation. What could be their function? One example is the VAMP-family proteins, controlling migration speed in CRs, as early as E11, possibly through regulation of exocytosis, asymmetric membrane transport and/or endosomal recycling. This function turns out to be dramatically important for regulation of cortical arealization during postnatal development, specifically for primary and secondary sensory cortices and its connection routing (Barber et al., 2015). Another example comprises the whole class of trans-synaptic cell-adhesion molecules (CAMs). CAMs are likely at the basis of primary organization of synaptic junctions, but along with that they are as numerous and efficient for synaptogenesis as they are multifunctional outside this process. CAMs families such as LAR-type RPTPs and their ligands, Slitrks, Cadherins, Teneurins, and Ephrins/Eph receptors are all involved in neuronal morphogenesis, both dendritogenesis and axonal pathfinding (Südhof, 2018). Interesting confluence of CAMs role on both morphogenesis and organization of electrical activity zones, such as the synapses, is reminiscent of gradual Ca^2+^ transients rise and morphological refinement of maturing migrating neurons. Few examples of CAMs regulation of neuronal migration exist (Qu and Smith, 2004; Kirkham et al., 2006; Funato et al., 2011; Sentürk et al., 2011; Puehringer et al., 2013; del Toro et al., 2017, 2020), however, whether the mechanisms involve electrical activity regulation remains an open question.

## Conclusion

In this review, we have discussed the distribution of ion channels canonically involved in the synaptic ion exchange machinery, in the attempt to decipher the principles of electric regulation of migration and maturation in early cortical development, before functional synaptogenesis occurs. While not all neurotransmitter receptor systems involved in the maturation of developing pyramidal neurons were considered (such as purinergic and cholinergic systems), a basic coupling principle seems to predominate: channels underlining excitatory responses and thus electrical cellular activation (with Ca^2+^ transients as a readout) contribute to migration pausing, eventual arrest and subsequent dendritogenesis (Table 1). Although it is hard to clearly correlate particular channels presence with dendritogenesis *vs.* migration arrest, progressive enrichment of specifically the NR2B subunit of NMDA receptors as well as the appearance of fast excitatory transmission supplied by AMPARs expression seem to be the most promising candidates for electrical regulation of initial neuronal maturation.

**TABLE 1:** Summary of ion channels having differential roles in migration and dendritogenesis of cortical glutamatergic neurons.

	Migration	Dendritogenesis	References
NMDA NR1	Yes	Basic expression levels are needed for proper dendrite arborization	Jiang et al., 2015
NMDA NR2B	Yes	Primary dendrite pruning and patterning	Espinosa et al., 2009; Jiang et al., 2015
AMPA GluR1, 2, 3	Yes*	Arborization length and complexity	Chen et al., 2009; Jansson et al., 2013; Hamad et al., 2014
NKCC1	No	Possibly. Effects on dendritic arborization in young hippocampal granule cells	Ge et al., 2006; Jiang et al., 2015
KCC2	No	Premature expression disrupts dendritogenesis	Cancedda et al., 2007
Kir2.1	No*	Premature expression disrupts dendritogenesis	Cancedda et al., 2007
SCN3A	Yes	Pathological variant attenuates dendritogenesis, but not migration	Smith et al., 2018
KCNK9	Yes	No	Bando et al., 2014

*Alternative interpretations still possible (see text).

Dendritogenesis is concurrent with synaptogenesis, and according to the synaptotrophic hypothesis, synaptogenesis comes first and is initially required for filopodia stabilization. The very first step in this process is recruitment of CAMs which subsequently leads to gradual synapse formation and dendritic stabilization and outgrowth (Chen and Haas, 2011). It is now explicitly demonstrated that functional synaptic contacts in the developing cortex help migration pausing and morphological reorganization during the multipolar-bipolar transition in proximity of the electrically mature zone, the SP (Ohtaka-Maruyama et al., 2018). However, how advanced the synaptic machinery assemblage must really be in order to assert proper electrical regulation of migration and maturation processes is unclear. As well as neurotransmitters and their receptors, CAMs expression throughout the early developing cortex is abundant and there are examples where partners for synaptic binding are expressed in complementary manner in areas formally devoid of synaptic structures. Further investigations are needed to fully answer the intriguing questions of how the congregation of synaptic molecules regulates neuronal maturation, whether this relies on adhesive or electric-organizing properties of CAMs or their combination, and how the neuronal electrical and adhesive machinery cooperate during cortical development.

## Author Contributions

VM and AP conceived and wrote the review. Both authors contributed to the article and approved the submitted version.

## Conflict of Interest

The authors declare that the research was conducted in the absence of any commercial or financial relationships that could be construed as a potential conflict of interest.
